# *rmtA*-Dependent Transcriptome and Its Role in Secondary Metabolism, Environmental Stress, and Virulence in *Aspergillus flavus*

**DOI:** 10.1534/g3.119.400777

**Published:** 2019-10-10

**Authors:** Timothy Satterlee, Sarah Entwistle, Yanbin Yin, Jeffery W. Cary, Matthew Lebar, Liliana Losada, Ana M. Calvo

**Affiliations:** * Department of Biological Sciences, Northern Illinois University, DeKalb, IL,; † Food and Feed Safety Research Unit, USDA/ARS, Southern Regional Research Center, New Orleans, LA, and; ‡ Infectious Diseases Program, J. Craig Venter Institute, Rockville, MD

**Keywords:** Aspergillus flavus, Environmental stress, RmtA, Secondary metabolism, Transcriptome, Virulence

## Abstract

*Aspergillus flavus* colonizes numerous oil seed crops such as maize, peanuts, treenuts and cottonseed worldwide, contaminating them with aflatoxins and other harmful toxins. Previously our lab characterized the gene *rmtA*, which encodes an arginine methyltransferase in *A. flavus*, and demonstrated its role governing the expression of regulators in the aflatoxin gene cluster and subsequent synthesis of toxin. Furthermore, our studies revealed that *rmtA* also controls conidial and sclerotial development implicating it as an epigenetic regulator in *A. flavus*. To confirm this, we performed a RNA sequencing analysis to ascertain the extent of *rmtA*’s influence on the transcriptome of *A. flavus*. In this analysis we identified over 2000 genes that were *rmtA*-dependent, including over 200 transcription factor genes, as well as an uncharacterized secondary metabolite gene cluster possibly responsible for the synthesis of an epidithiodiketopiperazine-like compound. Our results also revealed *rmtA*-dependent genes involved in multiple types of abiotic stress response in *A. flavus*. Importantly, hundreds of genes active during maize infection were also regulated by *rmtA*. In addition, in the animal infection model, *rmtA* was dispensable for virulence, however forced overexpression of *rmtA* increased mortality with respect to the wild type.

*Aspergillus flavus* is an opportunistic plant pathogen of great economic importance that infects oil seed crops such as maize, peanuts, cotton and certain treenuts, and in the course, produces potent mycotoxins (Hedayati *et al.* 2007), including the highly carcinogenic aflatoxins (Sarma *et al.* 2017). Ingestion of aflatoxin contaminated crops can result in jaundice, edema of the limbs, pain, vomiting, necrosis, potentially acute liver failure and in rare cases death (Lancaster *et al.* 1961; CDC, 2004; Fung and Clark 2004; Lewis *et al.* 2005). Chronic exposure can lead to suppression of the immune system, stunting of growth and wasting in children and several types of cancers such as those affecting the liver, lungs and gastrointestinal tract (CDC, 2004; Lewis *et al.* 2005; Marchese *et al.* 2018). In developed nations, legislation regulates levels of aflatoxins in food and feed commodities to prevent adulterated crops from entering the market place, however in most developing nations lacking such guidelines or restrictions, exposures becomes more prevalent (Ojiambo *et al.* 2018).

In the United States and other developed nations, the major impact of aflatoxin contamination of commodities is economic losses. It has been estimated that economic losses associated with aflatoxin contamination of maize can reach up to a billion dollars annually in the United States alone particularly in years with warm summers and drought (Mitchell *et al.* 2016).

In addition to its devastating effect on crops of economic importance, *A. flavus* is known to cause a deadly lung infection known as invasive aspergillosis. Although *A. flavus* is the second leading cause of IA after *Aspergillus fumigatus*, *A. flavus* is 100-fold more virulent than that of *A. fumigatus* (Ford and Friedman 1967; Mosquera *et al.* 2001; Kamai *et al.* 2002, Kaliamurthy *et al.* 2003 Hedayati *et al.* 2007).

Due to the adverse health and economic impacts associated with aflatoxin contamination and *A. flavus*, it is paramount to gain insight into its dispersal and survival mechanisms, as well as the regulatory pathways controlling its production of mycotoxins and its virulence. This knowledge could reveal novel genetic elements that could be used as possible targets to reduce the negative effects of this opportunistic pathogen of humans and plants.

Morphological development and secondary metabolism (SM) are genetically linked in *A. flavus* and other fungal species (*i.e.*, Calvo *et al.* 2002; Calvo and Cary 2015). In *A. flavus*, one of those genetic links is *rmtA*, encoding an arginine methyltransferase that has been shown to regulate aflatoxin biosynthesis as well as development (Satterlee *et al.* 2016). Specifically, *rmtA* is a repressor of the production of conidia, air-borne asexual spores that constitute an efficient form of fungal dissemination, and a positive regulator of sclerotial production, resistant structures that can survive under adverse environmental conditions (Horn *et al.* 2014; Satterlee *et al.* 2016). Homologs of RmtA have been shown to be involved in transcriptional regulation, signal transduction, RNA processing and transport (Bedford and Clarke 2009). RmtA is known to have a role in methylation of histones, which in turns affects gene expression (Trojer *et al.* 2004; Tessarz & Kouzarides 2014).

As *rmtA* appeared to be functioning as a global regulator of secondary metabolism and development, our current study was performed to further assess its influence on the transcriptome of *A. flavus*. In this analysis we identified over two-thousand genes that are *rmtA*-dependent, some of those genes are associated with secondary metabolism, abiotic stress response and virulence of this agriculturally and medically important fungus.

## Materials and Methods

### Strains used and growth conditions

All strains used in this work are listed in Table 1. Strains were grown on potato dextrose agar (PDA) at pH 5.6 in the dark at 30°, unless otherwise stated. Stocks of each strain were maintained as conidia at -80° in 30% glycerol.

**Table 1:** Strains used in this study

Strain	Pertinent Genotype	Source
CA14-WT	Δ*ku70*	USDA
CA14-Δ*rmtA*	Δ*rmtA*::*pyrG^A^*^.fumigatus^, *niaD^A^*^.fumigatus^, Δ*ku70*	Satterlee *et al.* 2016
CA14-com*rmtA*	Δ*rmtA*::*pyrG^A^*^.fumigatus^, *rmtA*::niaD*^A.fumigatus^*, Δ*ku70*	Satterlee *et al.* 2016
CA14-OE*rmtA*	*gpdA*(p)::*rmtA*::*trpC*(t)::*pyrG*^A.fumigatus^, *niaD*^A.fumigatus^, Δ*ku70*	Satterlee *et al.* 2016
CA14	*pyrG*-, *niaD*-, Δ*ku70*	USDA
CA14- *pyrG-1*	*pyrG+*, *niaD*-, Δ*ku70*	USDA
CA14- Δ*gliP*	Δ*gliP*::*pyrG^A.fumigatus^*, *niaD*-, Δ*ku70*	This Study
CA14- Δ*rmtA*	Δ*rmtA*::*pyrG^A.fumigatus^*, *niaD*-, Δ*ku70*	Satterlee *et al.* 2016

### Purification of RNA and sequencing

The wild type (WT), deletion *rmtA* (Δ*rmtA*), and overexpression *rmtA* (OE) strains were grown on potato dextrose top-agar in the dark at 30°. Spores (5x10^6^) were inoculated into 5 ml of melted PDA top agar (0.5%), which was then placed onto 25 ml solid PDA medium. After 72 h of incubation, mycelia was collected, frozen in liquid nitrogen, and lyophilized. Total RNA was extracted from mycelia using an RNeasy Plant Mini Kit (Qiagen, Germantown, Maryland, USA) following the manufacture’s protocol. RNA was further purified using Dynabeads mRNA Purification Kit. RNA quality was assessed using an Agilent Bioanalyzer. Sequencing was performed as a HiSeq 2000 single read 1x100bp lane. The experiment was carried out with 2 biological replicates.

### Analysis of RNA-sequencing data to identify differentially expressed genes

#### Read mapping:

The single-end reads of the WT, Δ*rmtA*, and OE samples were separately aligned to the *A. flavus* NRRL 3375 reference genome using HISAT2 (Kim *et al.* 2015) version 2.0.5. The command used was hisat2 -x reference_genome_index –U fastq_file -S output_file.sam. HISAT2 utilizes Bowtie2 (Langmead & Salzberg 2012) and was run using software version 2.3.HISAT2 utilizing Bowtie2 version 2.3.1. SAMtools version 1.3.1 was implemented to convert the SAM output file from TopHat into a BAM file for the next step.

#### Read counts:

The mapped reads in BAM format were then analyzed using the HTSeq.scripts.count command from the HTSeq python package version 0.9.1. This tool was employed to return a table of read counts for each gene. The command used was python –m HTSeq.scripts.count –i Parent gff_file. The GFF file downloaded from NCBI contains the pre-annotated gene models as well as their genomic locations.

#### Differential expressed genes (DEGs):

The table of read counts was used as input for the R limma package. This package was used to determine DEGs by comparing read counts between two conditions: WT *vs.* Δ*rmtA* and WT *vs.* OE. The two replicates of each condition were combined during this step of the analysis. The RPKM function in the R edgeR package determined the RPKM (reads per kilobase per million) values for all the genes. Bash and Perl scripts were developed to parse the DEGs data and RPKM data. An Excel file was created with the RPKM values for all genes across all conditions. FungiDB (Basenko *et al.* 2018) was used for functional enrichment of the data sets using Go Term (GO) annotations.

#### Selected groups of genes:

To gain more biological significance from the data sets, the differentially expressed genes were mapped to other databases. The list of SM gene clusters (SMGCs) information was extracted from Ehrlich and Mack (2014). A full list of transcription factors (TFs) in *A. flavus* was derived from the Fungal Transcription Factor Database (http://ftfd.snu.ac.kr/intro.php) (Park *et al.* 2008). Functional annotations of these transcription factors were obtained from NCBI. Genes related to environmental stress response were extracted from the database established by Miskei *et al.* 2009. The list of DEGs from the study performed by Dolezal and collaborators (Dolezal *et al.* 2013) was compared to this dataset to search for potentially *rmtA*–dependent virulence genes. R (R Core Team 2017) version 3.4.1, specifically the ggplot2 package (Wickham 2009), was used to make statistical figures.

### Analysis of novel epidithiodiketopiperazine cluster

#### Generation of gliP deletion strain (ΔgliP):

To impair the function of *gliP* part of the gene was knocked out. First, the *gliP* deletion cassette was created by fusion PCR as described by Szewczyk *et al.* (Szewczyk *et al.* 2006). Primers AFLA_gliP_P1 and AFLA_gliP_P2 were used to PCR amplify the 5′ UTR of the *gliP* locus in the *A. flavus* genome, while AFLA_gliP_P3 and AFLA_gliP_P4 primers were used to amplify the 3′ end of the *gliP* coding region. The middle fragment containing the *pyrG* selection marker was PCR amplified from the genomic DNA of *Aspergillus fumigatus* using primers AFLA_gliP_P5 and AFLA_gliP_P6. The three fragments were then fused by PCR using primers AFLA_gliP_P7 and AFLA_gliP_P8. All primers used in this study are listed in Table 2. The fused PCR product was transformed into *A. flavus* CA14 host strain (*pyrG*-, *niaD*-) by a polyethylene glycol-mediated transformation as previously described (Cary *et al.* 2014). Transformants were selected on half-strength PDA without uracil. Potassium chloride (0.6 M) was used as an osmotic stabilizer in the regeneration medium. Transformants were confirmed by diagnostic PCR with primers AFLA_gliP_P1 and Afum_pyrG_R. A selected *hbxA* deletion transformant, TTRS6, was used in this study.

**Table 2:** Primers used in this study

Primers	Sequence 5′ -> 3′
AFLA_gliP_P1	TCGATTCAGCGAGCCAGATGG A
AFLA_gliP_P2	GGAGGCTGCTTCAGGGTATAAGAGA
AFLA_gliP_P3	CCGATGCTTACCTGTGCTTTACGTC
AFLA_gliP_P4	GCCCAGTTGGAGGATATCACGGAA
AFLA_gliP_P5	TCTCTTATACCCTGAAGCAGCCTCCACCGGTCGCCTCAAACAATGCTCT
AFLA_gliP_P6	GACGTAAAGCACAGGTAAGCATCGGGTCTGAGAGGAGGCACTGATGCG
AFLA_gliP_P7	GGTGGATAACGGCAAGTC ATCTCCT
AFLA_gliP_P8	CGGCAATGAGATGGTTCCCCTG
Afum_pyrG_R	GAGCAGCGTAGATGCCTCGAC

#### Chemical analysis of ΔgliP, ΔrmtA, and WT strains:

*A. flavus* Δ*gliP*, Δ*rmtA*, and WT strains were cultivated in 1 L zeolite medium (glycerol 30 g/L, glucose 10 g/L, peptone 5 g/L, NaCl 2 g/L, molecular sieve 0.5 nm 10 g/L, agar 1 g/L, pH 7.0, see Chankhamjon *et al.* 2014) in 2.8 L Fernbach flasks at 28° with orbital shaking (110 rpm) for 4 days. The culture broth and mycelia were extracted with ethyl acetate (500 mL). The organic extracts were filtered through miracloth and concentrated to dryness under reduced pressure. Extracts were analyzed as described previously (Lebar *et al.* 2018) on a Waters ACQUITY UPLC system using PDA UV and QDa nominal mass detection [column: BEH C18 1.7µm, 2.1 × 50 mm; gradient solvent system: (0.5 mL/min, solvent A: 0.1% formic acid in water; solvent B: 0.1% formic acid in acetonitrile): 5% B (0-1.25 min), gradient to 25% B (1.25-1.5 min), gradient to 100% B (1.5-5.0 min), 100% B (5.0-7.5 min), then column equilibration 5% B (7.6-10.1 min)].

### Environmental stress assay

To assess *rmtA* involvement in response of *A. flavus* to environmental stresses, fungal strains were assayed with osmotic stress inducers using temperatures above and below optimum growth conditions. For all assays, WT, Δ*rmtA*, OE and the genetically complemented Δ*rmtA* strain (COM) were point-inoculated onto PDA plates supplemented with osmotic agents (1 M sucrose, 1.2 M sorbitol, 0.6 M KCl, or 0.7 M NaCl) and incubated in the dark for 48 h. For temperatures assays, cultures were exposed to 25°, 28°, 30°, 37°, 40° and 42°.

### Pathogenicity assay

Spores from WT, Δ*rmtA*, com, and OE strains were collected in a solution of 1x PBS with 0.1% tween and washed 5 times with additional equal volumes of 1x PBS. To reach the target concentration of 5x 10^3^ spores per 10 μl^-1^, the spores suspensions were diluted further with 1x PBS. The infection procedure was carried out as previously described by Fuchs *et al.* (2010). Briefly, *Galleria mellonella* larvae (The Bug Company, Ham Lake, Minnesota) with a weight range between 275–300 mg and lacking gray markings were selected for the experiment. Groups of 30 larvae were infected with the WT, Δ*rmtA*, OE, and complementation (COM) strains. An additional two group of 30 larvae each were used as controls. One group received injections of 10 µl of 1x PBS while the other groups received no injections. Larvae were then placed in glass petri plates (90 mm × 15 mm) and wrapped in aluminum foil. Plates were placed in 37° in the dark. Larvae were check every 4 h following 16 h of incubation until one group of larvae experienced complete mortality.

### Data availability

Table S1 contains calculated expression values comparing those of the wild-type strain to those of the deletion *rmtA* mutant and overexpression strain. Tables S2–S4 contain a subsets of Table S1 that includes DEGs corresponding to transcription factors, genes related to environmental stress, and genes that were shown to be DEG during maize infection. Figure S1 shows GO terms of DEG that are *rmtA*-dependent. Figure S2 details the strategy for the construction of the *gliP* mutant strain. Figures S3 and S4 show the *rmtA*-dependent effect of osmotic stress and temperature on *A. flavus*. The sequencing data are publicly available at NCBI’s SRA repository with the SRA Accession #: PRJNA573552. Supplemental material available at figshare: https://doi.org/10.25387/g3.9252530.

## Results

### rmtA-dependent transcriptome in A.flavus

**Figure 1:**
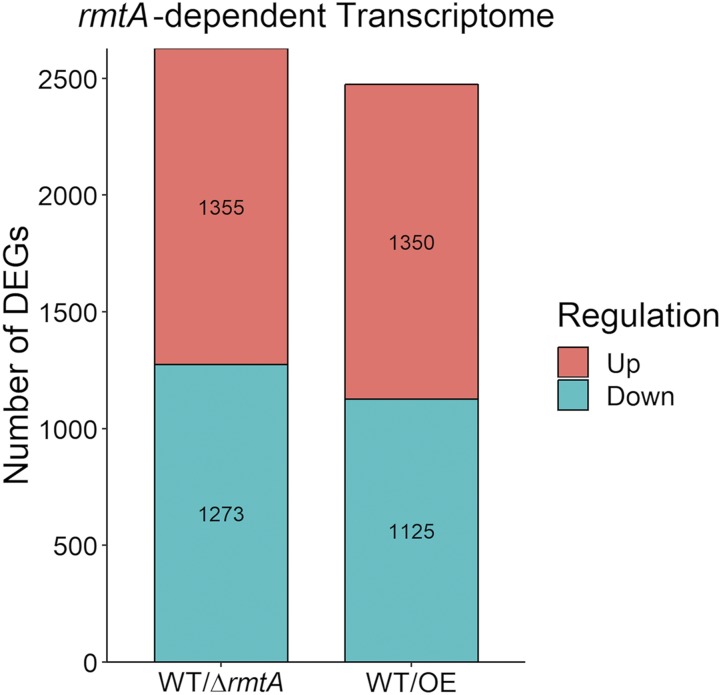
Number of upregulated and downregulated genes when the expression of *rmtA* is altered by *rmtA* deletion or overexpression. Number of up-regulated (red) and down-regulated (green) DEGs in Δ*rmtA*/WT and OE/WT comparisons.

RNA-seq analysis of the influence of *rmtA* on the *A. flavus* transcriptome revealed that both deletion or over-expression of *rmtA* results in similar ratios of up or down differentially expressed genes (DEGs) with greater than twofold difference in expression compared to the wild type. This constituted more than 2000 *rmtA*-dependent DEGs, as shown in Figure 1. Absence of *rmtA* affected the expression of more genes than when *rmtA* was over-expressed. Only 27 DEGs showed opposite expression patterns, DEGs that are downregulated in the absence of *rmtA* while they are upregulated when *rmtA* is overexpressed and vice versa. Most of the DEGs require wild-type levels of *rmtA* expression to function properly, as both deletion and overexpression of *rmtA* cause alterations in their transcription. There were 719 genes with reduced expression when *rmtA* was either knocked out or over-expressed, and 632 genes with increased transcription when this occurs. Full analysis is located in Table S1. Enrichment analysis of the functional categories of the *rmtA*-dependent transcriptome did not indicate any particular areas of regulation that *rmtA* governs either by its absence or forced expression (Figure S1).

### rmtA-Dependent Expression of SMGCs

**Figure 2:**
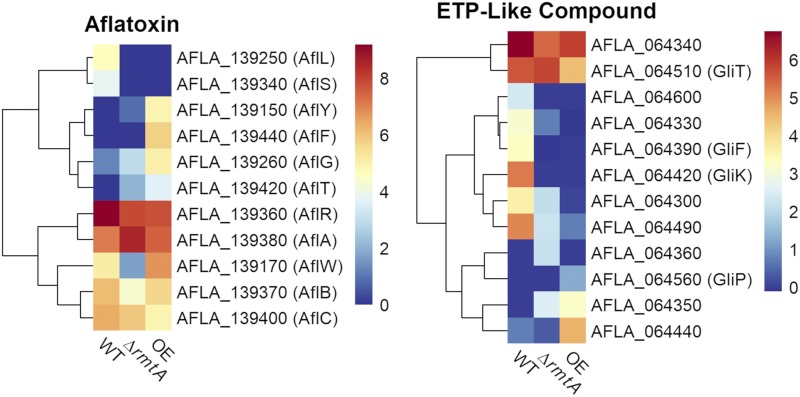
Heat maps of DEGs in Aflatoxin and epidithiodiketopiperazine - like compound gene cluster in the *rmtA*-transcriptome. Heat map of RPKM values of genes that are differentially expressed in *rmtA* transcriptome and found in the secondary metabolite gene cluster #54 Aflatoxin (left), and cluster #21 an uncharacterized cluster that may produce an epidithiodiketopiperazine (ETP) compound similar to gliotoxin (right).

Our results revealed that several genes within two secondary metabolite gene clusters (SMGCs) (defined in Georgianna *et al.* 2010) were *rmtA*-dependent (Figure 2). One cluster, denoted as #54, corresponds to the already characterized aflatoxin biosynthetic gene cluster. As shown previously, *rmtA* regulates production of aflatoxin (Satterlee *et al.* 2016). The other SMGC is the uncharacterized cluster #21 that contains several genes homologous to those associated with the gliotoxin gene cluster in *A. fumigatus*.

**Figure 3:**
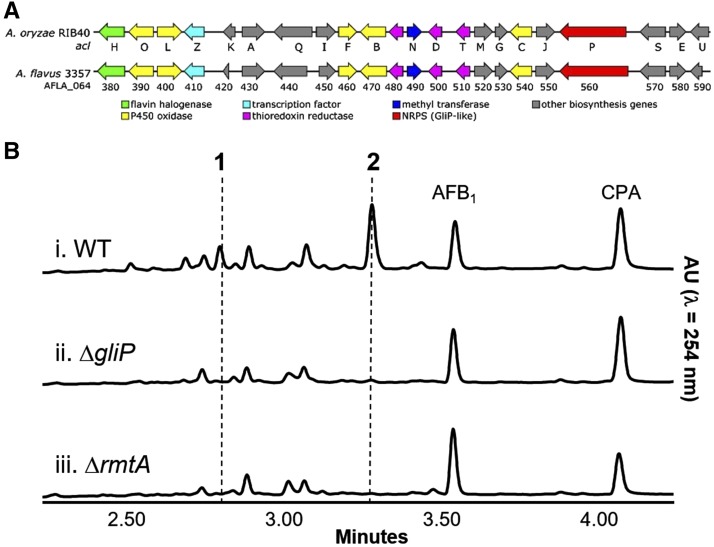
Chemical analysis of *A. flavus* strains. A) comparison of *A. oryzae* RIB40 aspirochlorine biosynthetic gene cluster *acl* and *A. flavus* 3357 biosynthetic gene cluster #21. B) UPLC chromatograms of *A. flavus* extracts: peaks **1** and **2** are present in WT (i), but not observed in Δ*gliP* (ii) or Δ*rmtA* (iii). CPA: cyclopiazonic acid, AFB_1_: aflatoxin B_1_.

In order to determine what metabolite(s) is the product associated with cluster #21 an *A. flavus* strain with a deletion of the putative nonribosomal peptide synthetase gene (AFLA_064560), homologous to that of *A. fumigatus gliP*, was created (Figure S2). This strain was confirmed by PCR, yielding the expected 3.1 kb PCR product. Cluster 21 bears considerable resemblance to *acl* (Figure 3A), the cluster responsible for aspirochlorine biosynthesis in *A. oryzae* (Chankhamjon *et al.*, 2014). However, initial chemical analysis of Δ*gliP*, Δ*rmtA*, and WT strains grown in PDA did not reveal any differences in secondary metabolite production, nor was aspirochlorine detected in any strains (data not shown). Chankhamjon *et al.* (2014) found that *A. oryzae* grown in optimized “zeolite” medium resulted in consistent aspirochlorine production. When *A. flavus* Δ*gliP*, Δ*rmtA*, and WT strains were cultured in zeolite medium, two peaks were observed in the WT extract (Figure 3B, i) that were absent in both Δ*gliP* (Figure 3Bii) and Δ*rmtA* extracts (Figure 3Biii). Neither of these peaks appear to be aspirochlorine, but may be biosynthetic intermediates or related epidithiodiketopiperazine analogs. The compounds ionized in negative mode (peak 1: [M-H]^-^ =455 *m/z*; peak 2: [M-H]^-^=419 *m/z*) and have UV λ_max_ = 230 nm.

### rmtA-dependent transcription factors

**Figure 4:**
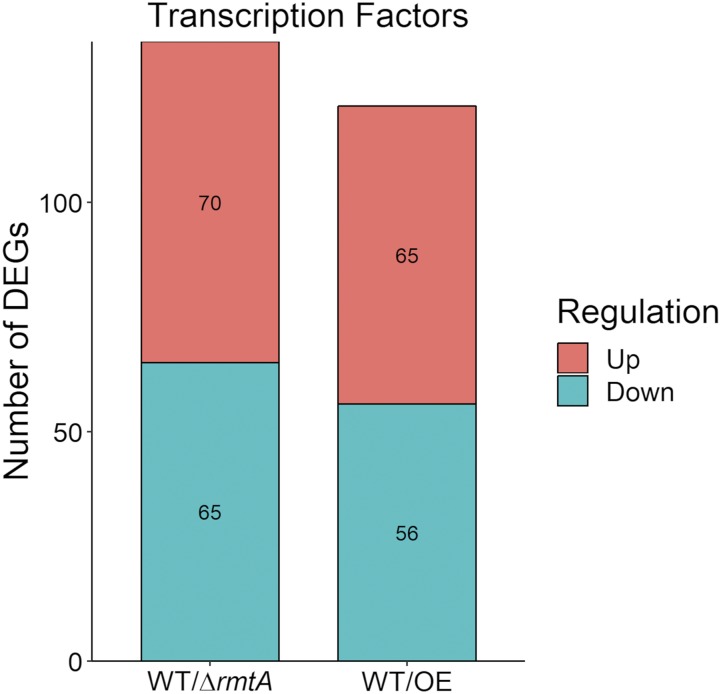
*rmtA*-dependent transcription factor genes. Graphical representation of *rmtA*-dependent transcription factor genes. The numbers of differentially expressed transcription factor genes whose expression is affected by either knock out or over-expression of *rmtA*. Red color indicates upregulated genes while green color indicates genes that are downregulated. A full list of transcription factors (TFs) in *A. flavus* was derived from the Fungal Transcription Factor Database (http://ftfd.snu.ac.kr/intro.php) (Park *et al.* 2008).

Based on our analysis, 251 out of the over 600 putative transcription factor genes in *A. flavus* were regulated by *rmtA* under the conditions used in the current study (Figure 4). Some of the transcription factors shown to be governed by *rmtA* are known to be involved in the regulation of development and metabolism, such as the aflatoxin transcription factor AflR (Woloshuk *et al.* 1994); MetR, a regulator of sulfur metabolism (Jain *et al.* 2018); and Rum1 which in *A. flavus* regulates both asexual development and metabolism (Hu *et al.* 2018). In addition, multiple transcription factors were previously shown to be associated with pathogenicity (Bultman *et al.* 2016; Issi *et al.* 2016). In other species genes such as *con7*, *ctf1*, *metR* and *sreA* were found to be connected with virulence, and their homologs are dependent on *rmtA* in *A. flavus* (Schrettl *et al.* 2008; Ramírez and Lorenz 2009; Ruiz-Roldán *et al.* 2015; Gai *et al.* 2019). Additionally, transcription factors attributed to different types of environmental stress response in *Aspergillus* were also found to be regulated by *rmtA* such as SrrA (oxidative and osmotic; Hagiwara *et al.* 2011), HacA (thermal; Zhou *et al.* 2016), AtfA (oxidative and osmotic; Balázs *et al.* 2010), and Seb1 (osmotic, oxidative, and thermal; Seidl *et al.*, 2004). A complete list of *rmtA*-dependent transcription factors are located in Table S2.

### rmtA Acts as a Repressor of Conidiation even under Environmental Stress

**Figure 5:**
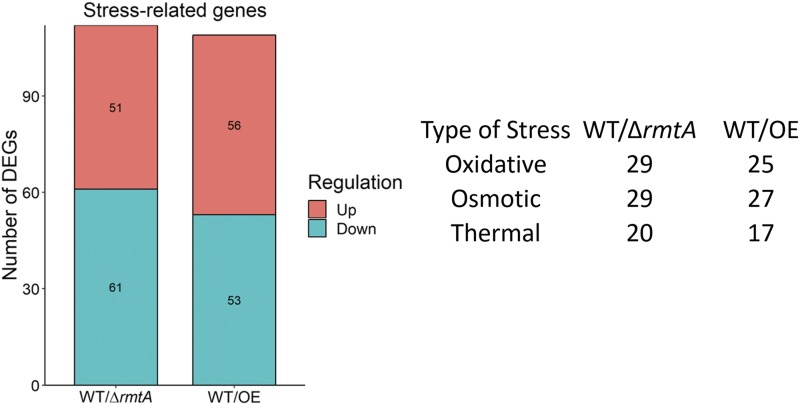
Impact of *rmtA* on the expression of stress-related genes in *A. flavus*. Graphical representation of the number of *rmtA*-regulated stress response genes based on the Miskei *et al.* (2009) database.

The identification, in the current study, of stress response transcription factors that are regulated by *rmtA*, led us to further investigate whether other genes involved in fungal stress response are also under *rmtA* control. A list of gene associated with stress response from Miskei *et al.* (2009) was used to parse our transcriptome data. The absence or over-expression of *rmtA* resulted in expression levels of approximately 100 genes significantly deviating from that of the wild type and were related to stress response (Figure 5). Analysis of the expression data indicated that *rmtA* does not regulate genes responding to a single type of stress but rather affects the expression of genes responding to multiple types of stress (Table S3).

Whether *rmtA* plays a role in resistance to oxidative stress in *A. flavus* was previously assessed and it was found that when exposed to increasing concentrations of the oxidant menadione, alterations in *rmtA* expression (by deletion or overexpression of *rmtA*) improved resistance of *A. flavus* to this stress condition (Satterlee *et al.* 2016). However, the possible implications of *rmtA* on the effect of other environmental stresses have not been studied. Based on our findings that expression of several genes involved in osmotic and thermal stress response are influenced by *rmtA*, we examined whether *rmtA* is involved in resistance to those environmental stresses. Unlike under oxidative stress conditions, vegetative growth was slightly reduced in the absence of *rmtA* when cultures were exposed to high concentrations of NaCl (Figure S3A). However, colony growth was not affected when the strains were grown on high concentrations of sucrose, sorbitol or KCl. The hyperconidation phenotype of the deletion mutant was still detected even in the presence of high osmotic stress, although it was significantly attenuated.

In contrast to exposure to oxidative or osmotic stress, changes in expression of *rmtA* over a range of incubation temperatures did not cause any alterations in colony growth. While no change in growth was observed, the deletion mutant hyperconidiation phenotype persisted at all temperatures tested, except 42° (Figure S4).

### rmtA regulates genes that are active during colonization of live plant tissue

**Figure 6:**
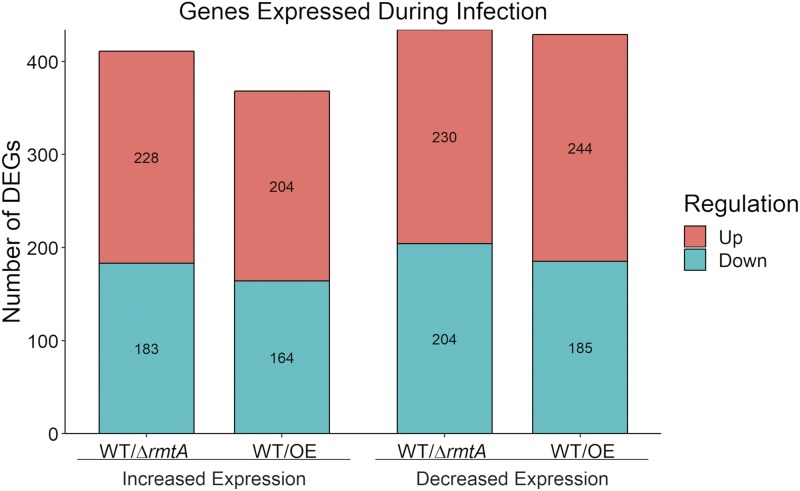
Effect of *rmtA* on genes active during plant infection. Graphical representation of *rmtA*-dependent genes that are differentially expressed during maize infection. This graph represents transcription factor genes whose expression is affected by either loss of or forced expression of *rmtA*. Red color indicates upregulated genes while green color indicates genes that are downregulated. The two left columns represent genes with increased expression during infection of plant tissue, while the two columns on the right indicate genes with decreased expression under that condition.

Dolezal *et al.* (2013) performed a transcriptome analysis of *A. flavus* during infection of maize that identified numerous DEGs during active infection *vs.* saprotrophic growth of this fungus using viable and nonviable maize kernels. The list of DEGs from the study performed by Dolezal was compared to our RNA-seq dataset to search for potential *rmtA*–dependent virulence genes. As shown in Figure 6, multiple genes that were differentially expressed during maize infection were also *rmtA*-dependent in the current experiment. Any modification of *rmtA* expression resulted in a decrease in the expression of 96 genes that were previously shown to be upregulated during maize seed infection. Conversely, we found 118 genes upregulated by changes in the *rmtA* locus that were suppressed during the seed infection study. A full list of genes that are regulated by *rmtA* and differentially expressed during plant infection are located in Table S4.

### Overexpression of rmtA increases virulence in Galleria mellonella animal model

**Figure 7:**
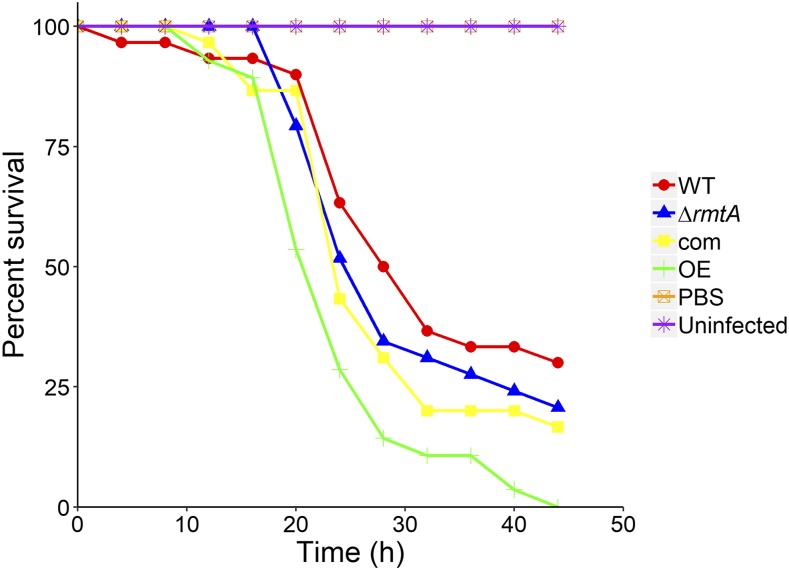
Overexpression of *rmtA* positively regulates virulence of *A. flavus* in the *G. mellonella* model. *Galleria mellonella* larvae were infected with *A*. *flavus* wild type (WT), deletion (Δ*rmtA*), complementation (com), and overexpression (OE) strains as described in Material and Methods section. Two controls groups were used in this experiment, one received an injection of 1x PBS instead of fungal spores, and another received no injection. Statistical analysis of survival was carried out by a Kaplan-Meyer pairwise comparison using a long rank test.

*Aspergillus flavus* is known to cause invasive aspergillosis in humans and animals. Since *rmtA* affects *A. flavus* infection in plants (Li *et al.* 2017), and our current study indicate that numerous genes active during plant infection are *rmtA*-dependent, we investigated whether *rmtA* also influences animal virulence. *Galleria mellonella* larvae were infected with WT, Δ*rmtA*, com, and OE strains to ascertain whether changes in *rmtA* expression affect virulence. While the deletion mutant showed a decrease in pathogenicity in maize and peanut infections, *rmtA* was dispensable for virulence in the animal model (Figure 7). We observed that overexpression of *rmtA* increased mortality in this model when compared to the control.

## Discussion

A comparative transcriptomic study of *A. flavus* control and *rmtA* mutants has provided further insights into the role of *rmtA* in the biology of *A. flavus*. Previously, it was shown that *rmtA* was a regulator of conidial and sclerotial production, as well as aflatoxin biosynthesis (Satterlee *et al.* 2016). Specifically, significant *rmtA*-dependent downregulation in expression of the conidiophore pathway developmental regulator *brlA* was shown to be the reason for reduction of conidiation in *A. flavus rmtA* knockout mutants. In terms of sclerotial production, knockout of *rmtA* halted production of these structures whereas increased levels of *rmtA* increased production compared to the wild type. RmtA was identified as positive regulator of aflatoxin production by directly affecting expression of aflatoxin cluster biosynthetic genes (Satterlee *et al.* 2016). Our present study revealed a broad regulatory scope for *rmtA*, where a significant portion of the *A. flavus* transcriptome is under its control. Hundreds of genes displayed altered expression upon comparison of expression levels in the wild type, Δ*rmtA* or overexpression *rmtA* strains; 719 genes in the *A. flavus* genome showed a reduction of their expression with this criterion, while 632 genes experienced an increase. In the model fungus *A. nidulans*, the *rmtA* homolog presented strong specificity for the methylation of histone H4 (Trojer *et al.* 2004). Epigenetic modifications of histone cores, such as histone methylation, affect nucleosome structures, leading to changes in the transcription of numerous genes (Tessarz and Kouzarides 2014). This agrees with the extensive effect of *rmtA* on the *A. flavus* transcriptome. In addition, based on our results, a balanced stoichiometry of RmtA with other partners seems to be required for its proper function.

As mentioned above, *rmtA* was found to be necessary for production of aflatoxin (Satterlee *et al.* 2016). Our transcriptome analysis revealed that out of 24 genes present in the aflatoxin SMGC (cluster #54 as in Georgianna *et al.* 2010), 11 genes were found to be *rmtA*-dependent. Outside of the aflatoxin cluster, only one other cluster, #21, demonstrated a large number of *rmtA*-dependent DEGs. In *A. flavus* cluster #21 has yet to be characterized. However, some genes in this cluster have homology to genes in the gliotoxin cluster in *A. fumigatus* (Dolan *et al.* 2015). Although there are similarities between these two clusters, the predicted cluster in *A. flavus* has nearly double the number of genes compared to that in the *A. fumigatus* cluster. Gliotoxin belongs to a class of metabolites known as epidithiodiketopiperazine. In *Aspergillus oryzae*, a cluster akin to the one described in *A. flavus* was characterized and found to produce another compound in this same family known as aspirochlorine (Chankhamjon *et al.* 2014). Production of this compound has been previously shown in *A. flavus* and documented to possess antifungal properties (Klausmeyer *et al.* 2005). Aspirochlorine was not detected in our *A. flavus* strain. However, chemical analysis of *A. flavus* WT and mutants did reveal two compounds present in WT that were not produced in SMGC#21 NRPS deletion mutant or the mutant lacking *rmtA*. Research on whether SMGC#21 is responsible for the synthesis of aspirochlorine or related epidithiodiketopiperazine compounds in *A. flavus* is ongoing. Surprisingly, levels of AF in Δ*rmtA* were similar to those in WT when the strains were grown in the zeolite medium, while production of this toxin was inhibited when the Δ*rmtA* strain was cultured on PDA (Satterlee *et al.*, 2016), suggesting that the effect of *rmtA* on AF production is medium-dependent.

While methylation of histones by *rmtA* may directly regulate the expression of certain genes in the genome, it would also affect the transcription of others indirectly, including transcription factor genes. In our study we identified over 200 transcription factor genes with altered expression patterns caused by by either deletion or overexpression of the *rmtA* locus. While they are not all functionally characterized, some of these transcription factors are known to play crucial roles in fungal development, metabolism, response to environmental stresses, and virulence. A few examples of these genes investigated in *A. flavus* include *aswA*, a regulator of sclerotial production and related metabolism (Chang *et al.* 2017), and *aflR* which is the primary regulator of aflatoxin production in *A. flavus* (Masanga *et al.* 2015). Another example is *rum1*, a transcription factor that has a wide range of regulatory effects, controlling aflatoxin biosynthesis, and development of conidia and sclerotia in *A. flavus* (Hu *et al.* 2018). However, the majority are still uncharacterized or have been studied in other fungi such as the medusa transcription factor MedA which is shown to regulate conidiation in multiple fungi (Clutterbuck 1969; Chacko and Gold 2012; Gravelat *et al.* 2010).

Unexpectedly, the *A. flavus* Δ*rmtA* mutant is more resistant to sources of oxidative stress than the wild type (Satterlee *et al.* 2016). This was in contrast with the phenotype of the Δ*rmtA* mutant in *A. nidulans* (Trojer *et al.* 2004), suggesting a specialization in the regulatory output of *rmtA* in both fungi with respect to environmental stress resistance. In our transcriptome analysis several genes such as *atfB* (Sakamoto *et al.* 2008), *fhdA* (Malavazi *et al.* 2006), *alb1* (Tsai *et al.* 1998), and *pes1* (Reeves *et al.* 2006) were found to be upregulated in the absence *rmtA*. Expression of these genes has been shown to be indispensable for resistance to oxidative stress. It is possible that the effect of *rmtA* on environmental stress resistance could be mediated by its effect on the expression of these genes.

Furthermore, we identified additional *rmtA*-dependent genes involved in response of the fungus to other environmental insults, such as those involved in osmotic and thermal stress. Examples of these genes include members of the HOG pathway, *nikA* and *shoA*, a well-studied network that regulates osmotic stress in fungi (Furukawa *et al.* 2005; Hagiwara *et al.* 2013). Also, *hacA* and *cypB* are *rmtA*-dependent DEGs where HacA is a heat shock protein and *cypB* is expressed at high levels during heat shock conditions (Joseph *et al.* 1999; Zhou *et al.* 2016). Based on these transcriptome results, we also examined whether *rmtA* influences the growth of *A. flavus* colonies when challenged by osmotic or thermal stresses. In most cases no changes in vegetative growth were detected. Only high concentrations of NaCl resulted in a slight growth reduction compared to the wild type under the same experimental conditions, suggesting an effect of *rmtA* on sodium metabolism. Although some stress response genes were affected by alterations in *rmtA* expression, fungal colony growth was not notably changed, which suggests possible redundancies in a robust genetic system in *A. flavus* protecting it from environmental stresses. Interestingly, the hyperconidiation phenotype of Δ*rmtA* persisted in the presence of the stressors assayed, and it was only partially attenuated under osmotic stress, suggesting that even under exposure to environmental stress *rmtA* is still a required regulator of asexual development in *A. flavus*.

Li *et al.* (2017) reported that *rmtA* affects development and aflatoxin production during infection of peanuts seeds and maize kernels (Li *et al.* 2017). While this study did not examine whether removal of *rmtA* influenced fungal burden during infection, *rmtA* was found to regulate lipase and protease activity (Li *et al.* 2017). In our transcriptome analysis we investigated connections related to virulence in genes regulated by *rmtA* based on a study of Dolezal *et al.* (2013), which identified DEGs during *A. flavus* infection of maize. Our study revealed that several DEGs encoding classes of secretory enzymes such as lipases (PlaA & PLD), proteases (Pim1 & MEP1) and several putative hydrolases (AFLA_025360, AFLA_004540, & AFLA_062930) (Hong *et al.* 2005; Brown *et al.* 2007; Zhang *et al.* 2014; Ciesielski *et al.* 2016) were *rmtA*-dependent. As mentioned earlier, a regulator of conidiation in aspergilli, MedA (Clutterbuck 1969), is regulated by *rmtA*, but it is also important in virulence, as it was shown to be required for biofilm formation and normal adhesion in *A. fumigatus* (Gravelat *et al.* 2010). Additionally, its homolog in *Ustilago maydis* is also necessary for full virulence in maize (Chacko and Gold 2012). Although *rmtA* is relevant in the colonization of oil seeds, our results indicate that this gene is dispensable for virulence in the *Galleria* animal model. Furthermore, elevated expression of *rmtA* caused an increase in mortality rate. Our transcriptome analysis indicated that genes involved in iron metabolism such as *sreA* (Schrettl *et al.* 2008), and *pes1*, which in *A. fumigatus* was shown to be necessary for full virulence in *G. mellonella* (Reeves *et al.* 2006). Both of these genes were upregulated by increased expression of *rmtA* and may, at least in part, contribute to the increased mortality observed in the overexpression strain.

In conclusion, we have shown that the epigenetic regulator *rmtA* governs the expression of over 2000 genes, affecting multiple aspects of *A. flavus* biology, including development and virulence in plants. It also regulates some aspects of environmental stress response and secondary metabolism, including an uncharacterized biosynthetic gene cluster that may be responsible for the production of an epidithiodiketopiperazine-like compound. It is interesting that although some of these genes have been previously characterized, the function of most *rmtA*-dependent genes remains unknown, constituting a new avenue to be further explored in future research. Importantly, although RmtA is well conserved in eukaryotes (Satterlee *et al.* 2016), the similarly is low at the N-terminal and C-terminal regions of this protein. These regions could be potentially used as a target to develop a strategy to reduce the detrimental effects of this agriculturally important fungus.
